# Influence of Multi-Role Interactions in Community Group-Buying on Consumers’ Lock-In Purchasing Intention From a Fixed Leader Based on Role Theory and Trust Transfer Theory

**DOI:** 10.3389/fpsyg.2022.903221

**Published:** 2022-06-15

**Authors:** Jingjing Wu, Yiwei Chen, Hao Pan, Anxin Xu

**Affiliations:** College of Economics and Management, Fujian Agriculture and Forestry University, Fuzhou, China

**Keywords:** role interaction of merchant, role interaction of friend, community group-buying trust, interpersonal trust, lock-in purchasing intention from a fixed leader

## Abstract

Community group-buying platforms are increasingly relying on the interaction between the group-buying leader and consumers, thereby achieving the customer lock-in. In view of this, it is crucial to understand how the group-buying leader to establish a long-term transaction relationship with consumers. In this study, we construct a model based on the role theory and trust transfer theory, and identify two types of interactions of the group-buying leader (i.e., role interaction of merchant and role interaction of friend) and two types of consumer trust (i.e., community group-buying trust and interpersonal trust). Then, the mechanism that how different role interactions of the group-buying leader can be transformed into the lock-in purchasing intention of consumers is further clarified. By interviewing 430 consumers with community group-buying experience in the community through offline questionnaire, the research model has been proven to be effective. To be specific, both role interactions of the merchant and friend can impose a positive impact on interpersonal trust, which will also lead to the trust in community group-buying, and thus enhance the lock-in purchasing intension of consumers from a fixed leader. Overall, this study has made certain contributions to the study of customer relationship. In theory, this study further explains the explanation mechanism of the “acquaintance marketing” phenomenon. Moreover, this study adopts the role theory to analyze the differences of different role interactions of the group-buying leader in relationship quality and purchase decision making, and employs the trust transfer theory to expand the trust transfer effect from the interpersonal trust of the group-buying leader to the trust in community group-buying. In practice, this study provides a new perspective and practical reference for community group-buying enterprises and the group-buying leader on how to better manage customers and maintain a long-term and stable customer relationship.

## Introduction

Group-buying is a marketing channel that gathers consumers through the network platform and stimulates consumers to buy products or services by providing a certain discount (Kao et al., 2020). It is widely used in retail, restaurant, hotel, and other industries. The traditional group-buying includes Meituan, Juhuasuan, Pinduoduo, and other group-buying service platforms. With the continuous development of fresh e-commerce, the community group-buying as a new group-buying mode combining online fresh e-commerce and offline community retail is launched. Some typical community group-buying platforms include Duoduo Buying, Meituan Optimization, Sincerity Optimization, and Taocaicai. They mainly rely on Internet community media channels (WeChat group), in which the group-buying leader acts as the head to organize and manage group members in the offline community, and regularly initiate the group-buying of “presale – next-day delivery – pick up” (Hao et al., 2020). Due to the influence of COVID-19, the business market of community group-buying in China develops very fast, and the increase rate of transaction size reached 300% in 2019. In 2021, the transaction size reached 120.51 billion CNY, and increased by 60.4% from the same quarter of last year. The user scale reached 0.646 billion people, with a growth of 37.44% year on year (WJS, 2022). Community group-buying effectively solves the problem of “logistics and distribution at the last-mile,” and becomes the new potential of digital economy development. However, with the rapid growth of the market and user scale, the stickiness of new users has become one of the practical problems that community group-buying platforms need to overcome urgently. The community group-buying leader and members live in the same community and communicate frequently no matter online or offline, and therefore, the members will long-term purchase products from the same leader due to the interpersonal relationship to a certain extent. This kind of stable purchase and sale relationship, or the consciousness or behavior that members long-term purchase products from the same leader under the context of acquaintances marketing, is defined as the lock-in purchasing behavior (Sun and Li, 2018). Therefore, it is of great significance to explore related research on the lock-in purchasing intention from a fixed leader, which is also deserved more attention by academic circles.

Community group-buying is considered a marketing activity based on interactions in the context of acquaintances. Scholars have studied the positive effect of interactions on purchasing behaviors based on the online interactions of online platforms and the interpersonal interactions of offline retailers (Li et al., 2019; Hussain et al., 2022; Li and Li, 2022). However, there are few studies on the influence of combined interactions featured by both online social networks characteristics and offline geographical scope characteristics on purchasing behaviors. Yan and Li (2021) pointed out that compared with virtual communities and traditional e-commerce, community group-buying supplies a convenient and real interpersonal interaction for the leader and members. In the context of community group-buying, the leader is the bridge to connect the platform and consumers, and the interactions between the leader and consumers throughout the entire online and offline purchasing process. Therefore, it is an important problem of community group-buying development to study the influence mechanism that interactions between the leader and members can affect consumers’ lock-in purchasing intention from a fixed leader.

Different types of interactions adopted by merchants have differentiated effects on consumers’ attitudes and behaviors (Fan et al., 2021). Heide and Wathne (2006) classified the roles that merchants played in the interactions into merchant and friend based on the role theory. KöHler et al. (2011) divided the interactions into task-oriented interaction and social-oriented interaction according to the features of interactions. This division is similar to the classification of merchant and friend based on the role theory. Due to the community group-buying involves online and offline service scenarios, the leader plays multiple roles in the interaction process with consumers, that is, they can shift in the role interactions between merchants and friends. Although the phenomenon of multi-role interactions has become more prominent in the development of emerging business models, the existing studies on the multiple roles that individuals may play in the interaction are rare, and there is less to explore the purchasing behavior according to different role interactions. In the mode of community group-buying, types of interactions may affect consumers’ lock-in purchasing intention from a fixed leader, and there may be differences in the effects of two different role interactions. For this reason, we introduce the role theory to explore why the topic of how different types of interactions affect consumers’ lock-in purchasing intention from a fixed leader has become the focus and research hotspot in academic circles.

In community group-buying, due to the separateness between consumers and goods, consumers cannot firsthand feel and identify whether the goods meet their expectations. In the context of e-commerce, trust is considered to be the key factor for effectively reducing uncertainty and risk perception (Li et al., 2020), and is also the key factor affecting the long-term purchase of consumers (Li et al., 2019; Jeon et al., 2021). Therefore, to establish long-term transaction relationships, merchants or platforms strive to adopt effective interaction strategies to build trust (Pang et al., 2021). The purchase situation of community group-buying extends from offline to online, and, in turn, the online supports the offline, leading to more complex trust construction than traditional trust. According to the classification of transaction subjects, community group-buying trust includes the double trust relationship between consumers with the group-buying leader and platforms (Fulmer and Gelfand, 2012). Moreover, it has been found that trust can be transferred between entities, and there exists a representative effect (Belanche et al., 2014; Konuk, 2020). To be specific, if an entity is the representative of other related entities, the consumer trust in this entity may also be assigned to related entities. In addition, trust is usually generated depending on the interactive environment and interactive identity of the trust subject (Su et al., 2021). Relying on the offline regional advantages, it is easy for the community group-buying leader to establish initial interpersonal trust with consumers through offline interpersonal interaction (Yan and Li, 2021). Based on the trust transfer theory, the interpersonal trust of the community group-buying leader plays a key role in building trust in the platform. Therefore, when the community group-buying leader becomes the representative of the community group-buying platform, whether the consumer trust in the platform can be established through the interpersonal trust accumulated by interacting with the leader and can affect the lock-in purchasing intention of consumers from a fixed leader deserve to be further studied.

Community group-buying platforms are increasingly relying on the interaction between the group-buying leader and consumers, thereby achieving the customer lock-in. Given the importance of the group-buying leader in helping platforms establish a long-term transaction relationship with consumers, this study focuses on the role of the community leader, and with the aim of explaining how different role interactions of the group-buying leader can be transformed into the lock-in purchasing intention of consumers. This study based on the role theory and trust transfer theory, to analyze and conclude the types of different role interactions between the community group-buying leader and members. Meanwhile, by introducing the interpersonal trust and community group-buying trust as mediators, we construct a research model to explore the internal mechanism of the influence of different role interactions on consumers’ lock-in purchasing behavior from a fixed leader in the context of community group-buying. From the theoretical view, this study further explains the explanation mechanism of the “acquaintance marketing” phenomenon, which is of certain significance to the expansion of marketing management theory. Specifically, this study adopts the role theory to analyze the differences of different role interactions of the group-buying leader in relationship quality and purchase decision making, and employs the trust transfer theory to expand the trust transfer effect from the interpersonal trust of the group-buying leader to the trust in community group-buying. From a practical point of view, this study provides a new perspective and practical reference for community group-buying enterprises and leaders to better manage associations and maintain long-term and stable customer relations, and also provides theoretical guidance for community group-buying enterprises to avoid performance risks caused by the interpersonal relationships of the group-buying leader.

## Literature Review and Theoretical Basis

### Community Group-Buying

Community group-buying is a new type of e-commerce business that integrates the characteristics of the community, social community, and group-buying. With the help of online social channels, community group-buying can more efficiently and conveniently provide group-buying products and services, including publicity, consultation, ordering, payment, return and exchange, etc. Relying on the offline community, and based on the characteristics of “acquaintances,” the community group-buying leader can conduct face-to-face daily communication and interaction with members at a deeper level, shorten the psychological distance with members, improve the relationship perception, and enhance the trust among each other (Li et al., 2020). There are some differences between community group-buying and the brand social community studied in the past. To be specific, the brand social community in the past refers to the frequent communication and interaction of members in a virtual network based on the same interests, values, cognition, and other factors (Lee et al., 2021). The community group-buying is more like a practical exploration of virtual space extending to offline entities. It refers to the group-buying activities carried out by the members in the community based on the common daily purchase needs and relying on the leader’s online WeChat group.

Overall, as an emerging social retail mode, community group-buying has attracted much attention in the recent 2 years, mainly focusing on the online and offline competition and fairness of community group-buying (Wang and Song, 2022), price strategy (Lin et al., 2020), online and offline channel interactivity (Cui et al., 2022), and consumers’ inventory behavior during the coronavirus outbreak (Hao et al., 2020). However, there is less attention focusing on the leader. The leader is an important group, and is the key entrance for the platform to seize community resources. The sources of the leader include mothers having babies, owners of convenience stores or express stations, property managers, and other people who have extensive contacts in the community networks (Yan and Li, 2021). Therefore, they can develop an efficient and stable way to expand customers.

### Lock-In Purchasing Intention

Lock-in theory comes from economics, and mainly discusses the reasons, mechanism, and influence of the phenomenon that consumers become long-term customers of a brand or a merchant (Janssen and Jager, 1999). Scholars mainly explored the influencing factors of the lock-in purchasing intention from the perspectives of online scenarios and offline scenarios. Based on the community environment, Sun and Li (2014) took the purchase of agricultural products as an example and revealed that the limited geographical scope and community interpersonal relationship increase the conversion cost of farmers, resulting in a stable purchase and sales state. In the network economy environment, although the open virtual Internet environment eliminates the restrictions of information access and geographical scope, the cost of channel conversion for users has not disappeared. The studies proposed that users form a lock-in effect on the platform due to the influence of early use, appreciation of cumulative information, learning cost, search cost, product compatibility, and other factors (Zhou and Zhao, 2017). The previous research on the scenarios of the lock-in purchasing intention was mostly the analysis of consumer behaviors under the background of a single offline community environment or online network economy. In view of the integration of online and offline market environments in community group-buying, the explanation of the lock-in purchasing intention from a fixed leader under the context of community group-buying needs to be further studied. In this study, based on the previous research and combined with the reality of community group-buying, we define the lock-in purchasing intention from a fixed leader as the trust of the community members to the leader based on the situation of acquaintance marketing and the relatively stable interpersonal relationship, which is characterized as the willingness to long-term patronize the business of a fixed leader.

### Role Theory

Role theory originated from drama, and then its research field gradually expanded to sociology to study the behavior characteristics of individuals in the group or social background (Berg et al., 1995). According to role theory, a role is usually defined as a series of behaviors to be expected during the process of individuals interacting with their social environment or others (Montgomery, 1998). The actions of each social role follow the behavior norms aroused by its specific situation, that is, the role setting can affect the pattern of interactive behaviors (Montgomery, 1998; Zhang et al., 2018). The role theory is widely used in organizational management, channel governance, and other fields. For example, the role of female chairman has an impact on the innovation of small and medium-sized enterprises (Anglin et al., 2022), and the role perception of retailers in channel marketing can affect the trust relationship (Shou et al., 2011).

Since friendship is often embedded in business relationships, Heide and Wathne (2006) combined with the “friend-merchant” model proposed by Montgomery (1998), and concluded that the two most typical roles in a transactional relationship are merchant and friend. The role of merchant follows the task-oriented guidance and is committed to maximizing the interests of consumers. The role of friend follows the social-oriented guidance to establish an interactive and friendly relationship between the two sides. In the transaction relationship, the two roles of the transaction subject may be awakened at the same time. However, in a specific time, the transaction subject only plays the dominant role and guides its interactive behaviors pattern (Haytko, 2004; Su et al., 2021). Overall, the role theory breaks the basic assumption of “unitary subject” in the previous studies that the transaction subject takes this well-established pattern for action. Specifically, the merchant starts from playing the role of “merchant,” and trust gradually accumulates and develops with the continuation of the transaction relationship (Zhang et al., 2016; Cai et al., 2022). In reality, at the beginning of relationship establishment, merchants may play the role of “friends” and keep a good personal relationship with consumers. Therefore, the two sides of the transaction may establish the business relationship based on the existing social relationship (Zhuang and Xi, 2003; Gao et al., 2016).

Studies have found that interactivity can affect the trust and community group-buying intention (Li et al., 2020). However, in community group-buying, the interaction pattern of the group-buying leader is closely related to its role attribute (merchant or friend). It is necessary to further analyze the differences of different role interactions of the group-buying leader in relationship quality and purchase decision making from the perspective of role theory. Therefore, referring to the views of Heide and Wathne (2006), from the perspective of identity interaction of different roles, this study divides the interaction between the leader and members in the community group-buying into two types, i.e., the role interaction of merchant and the role interaction of friend, and discusses the internal mechanism of the influence of different role interactions on consumers’ lock-in purchasing intention from a fixed leader. Specifically, the role interaction of merchant refers to the interactive form in which merchants display information, ability, skills, and efficiency to consumers, with the purpose of completing specific tasks, such as product information transmission, return and exchange, etc. The role interaction of friend refers to the interactive form in which the leader shows friendship, mutual assistance, and warmth to consumers, such as greeting in daily life; interaction in WeChat Moments and Escrow; and deposit items, etc.

### Consumer Trust and Trust Transfer Theory

In marketing management, trust is the friendly perception of consumers on the credibility of others’ speech and behavior in an uncertain environment (Mayer et al., 1995). The previous studies have shown that no matter online or offline, trust plays an important role in the long-term trading relationship (Jeon et al., 2021). Especially in the high-risk e-commerce situation, if the consumer trust is low, the transaction will not be successful (Hung et al., 2015).

Trust transfer theory indicates that the consumer trust in an unfamiliar subject is usually generated by transmitting the trust in other familiar subjects (Stewart, 2003). Trust transfer has been proved to be an effective mechanism for individuals to build and improve trust in different research fields. For example, Fu et al. (2015) indicated that when consumers are not familiar with an enterprise, the employee trust at the interpersonal level will affect the enterprise trust at the organizational level. Chen and Shen (2015) found that the consumer trust in the community members has a positive impact on the consumer trust in the social platform. Chen et al. (2015) showed that the credibility of the platform plays an important role in determining the trust of consumers in platform sellers. The recent studies have revealed that the offline trust of consumers will shift to online trust (Xiao et al., 2019; Jeon et al., 2021). This is due to that when consumers do not have direct information and sufficient experience about the online platform, the offline interaction with local store retailers can provide another information channel to enhance their trust in the platform.

Overall, the existing studies have explored the mechanism of trust transfer in the context of social e-commerce, but research on the mechanism that the offline trust transfers to the online trust is rare. Moreover, due to the complexity of the role and identity of the community group-buying leader, consumers have higher requirements for trust, leading to more complex and situational of the trust mode. Therefore, combined with the characteristics of community group-buying and the two roles of the leader, and by referring to the view of Fulmer and Gelfand (2012), this study divides consumer trust into community group-buying trust and interpersonal trust. Community group-buying trust refers to the belief of consumers in the attributes of integrity, service, and ability of the platform. Interpersonal trust refers to the belief of individuals in the attributes of integrity, service, and ability of the group-buying leader. Hence, to better understand the interaction between different trust in community group-buying, the trust transfer theory is employed to further study whether there is a trust transfer effect between interpersonal trust and community group-buying trust.

## Model Construction and Research Hypotheses

**FIGURE 1:**
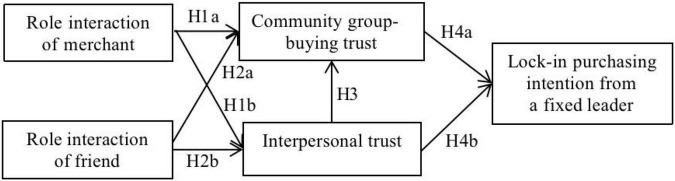
Conceptual model of the study.

In this study, by combining the role theory and trust transfer theory into an integrated analysis framework, we construct a conceptual model to explore the impact of different role interactions of the group-buying leader on the lock-in purchasing intention of consumers from a fixed leader in the community group-buying mode, as shown in Figure 1. First, based on the role theory, the impact of role interactions of merchant and friend on the community group-buying trust and interpersonal trust is studied. Second, based on the trust transfer theory, the trust transfer effect between interpersonal trust and community group-buying trust is investigated. Finally, the impact of interpersonal trust and community group-buying trust on the lock-in purchasing intention of consumers is analyzed. The following sections will elaborate on the assumptions of the research model.

### Role Interaction of Merchant vs. Community Group-Buying Trust and Interpersonal Trust

The virtualization characteristics of many new e-commerce transactions lead to large information asymmetry between merchants and consumers, and also improve consumers’ purchase cost and risk perception (Rosillo-Díaz et al., 2019). Empirical evidence shows that merchants can improve information asymmetry and effectively induce consumers to trust the capability of platforms through the interaction of informational content (Choi and Lee, 2016; Wang and Wang, 2022). In addition, some studies have shown that based on the interaction of emotional content, merchants can meet the interests and emotional demands of consumers, improve consumers’ perception of warmth and goodwill on merchants, and form interpersonal trust (Meire et al., 2019; Wang and Wang, 2022). Wang and Zhou took Airbnb as an example, i.e., a landlord sends out the signal of willingness to spend time on transactions by displaying social information to the consumer, indicating that the landlord’s attention to transactions can enhance the consumer’s trust in the landlord and affect the reservation intention of the consumer (Wang and Zhou, 2021). Based on the above research, when the community group-buying leader shows consumers information such as product production, purchase, and promotion through different interaction forms such as pictures, videos, and explanations in WeChat group or WeChat Moments, it will positively affect consumers’ trust in community group-buying; When the community group-buying leader establishes a good interaction relationship with consumers through timely communication and exchange of comments and timely handling of return and exchange requirements, it will positively affect consumers’ interpersonal trust in merchants. Therefore, the research Hypotheses H1a and H1b are put forward as follows:

H1a: In the context of community group-buying, the role interaction of merchants has a positive influence on the community group-buying trust.
H1b: In the context of community group-buying, the role interaction of merchants has a positive influence on the interpersonal trust.

### Role Interaction of Friend vs. Community Group-Buying Trust and Interpersonal Trust

Many new e-commerce enterprises face greater trust challenges in the process of development due to the lack of applicable market supervision and institutional mechanisms (Hu and Shu, 2021). The previous studies have shown that under this condition, the interaction between neighbors and friends in the social environment plays an important role in the participation and purchase decision making of consumers (Liang et al., 2020). According to the Elaboration Likelihood Theory, when it is difficult for consumers to judge the quality of the new and unfamiliar business, they will take the recommendation of trusted friends as the key factor in the peripheral path, and change their attitude to the new business and purchase decisions (Shahab et al., 2021). In the community, friendly interactions can be seen everywhere, such as greeting and asking for help. The more frequent this face-to-face interaction is, the easier it is to establish intimate interpersonal trust (Bosworth and Turner, 2018). With the rapid development of social media, the interaction between neighbors no longer depends on physical meetings (Bartal et al., 2019). Studies have proved that, unlike the strong relationship between relatives and friends, online community netizens generally have a weak relationship; however, merchants show a warm attitude toward each potential customer through the role of friends, such as giving thumbs-up, comments, or replies in their WeChat Moments, which will gradually turn the weak relationship to a strong relationship, and gradually form a positive judgment on the credibility of merchants and their product quality (Geng, 2021). Based on the above research, in community group-buying, the reliable image of the leader based on the role of friend will reduce the uncertainty of consumers, improve consumers’ attitude toward community group-buying, and have a positive influence on the trust of community group-buying. Meanwhile, the offline daily interaction will enhance the familiarity between the leader and members and positively affect the interpersonal trust. Therefore, the research Hypotheses H2a and H2b are put forward as follows:

H2a: In the context of community group-buying, the role interaction of friends has a positive influence on the community group-buying trust.
H2b: In the context of community group-buying, the role interaction of friends has a positive influence on the interpersonal trust.

### Interpersonal Trust and Community Group-Buying Trust

Trust transfer is an important characteristic of social commerce different from other traditional e-commerce. Based on the trust transfer mechanism, emotional trust will advance consumers to believe in the quality assurance of products sold by merchants (Lin et al., 2019). Chen et al. (2022) pointed out that with the increase of the consumer trust in the anchor of live e-commerce broadcast, their trust will transfer to the recognition of products and platforms. Su et al. (2021) took WeChat merchants as an example, and revealed that when consumers face multiple roles of merchants, consumers’ trust in the role of friends will affect their trust in the role of merchants and be regulated by the relationship. Based on the above research, when the community members believe that the leader is trustworthy, they will believe in the warmth and goodwill of the leader, and also believe that the leader has the ability to ensure the quality and after-sales of products, that is, the interpersonal trust can help to establish trust in the whole community group-buying. Therefore, the research Hypothesis H3 is put forward as follows:

H3: In the context of community group-buying, interpersonal trust has a positive influence on community group-buying trust.

### Consumer Trust and the Lock-In Purchasing Intention From a Fixed Leader

Consumer trust is an important concept in marketing research, which largely affects the online stickiness, repeated purchase intention, and other post-purchase behaviors of consumers in social media (Antwi, 2021; Zhang et al., 2022). Lee et al. (2021) pointed out that trust is the link between consumers and brand social communities. When consumers form a trusting attitude toward a brand social community, it will advance them to obtain a higher sense of security, thereby forming a tendentious purchase behavior for fixed brands. In addition, Molinillo et al. (2020) also verified that community trust is an important prerequisite for advancing consumers’ continuous participation in community activities. However, in the offline community retail environment, interpersonal trust plays a more important role. Consumers’ trust in fixed merchants due to the human relationship between neighbors will promote their high willingness to patronize (Li and Li, 2022). In the WeChat Moments marketing, consumers are more likely to purchase from a fixed merchant if the merchant maintains a stable interpersonal relationship with consumers (Geng, 2021). Based on the above research, with the accumulation of trading experience, consumers’ trust in community group-buying can help them avoid the uncertainty risk caused by changing other platforms, and form a willingness to maintain long-term trading with a fixed leader. When consumers incorporate the leader into a relatively close circle of acquaintances, a strong relationship trust is established, and this interpersonal trust is easier to maintain the long-term transaction. Therefore, the research Hypotheses H4a and H4b are put forward as follows:

H4a: In the context of community group-buying, the community group-buying trust has a positive influence on the lock-in purchasing intention from a fixed leader.
H4b: In the context of community group-buying, the interpersonal trust has a positive influence on the lock-in purchasing intention from a fixed leader.

## Research Design

### Concept Measurement

Based on the research background of community group-buying, this study refers and adapts the previous relevant maturity scale in the items involving five constructs in the research model, and uses 7-point Likert Scale for measurement. In the measurement, 1 indicates completely disagree and 7 indicates completely agree.

The role interaction of merchant (RM) is adapted based on the research of Williams and Spiro (1985), and there are three items to be measured, including RM1, the leader actively provides members with information about product production, purchase, quality, etc., RM2, the leader responds to the inquiries of members in time, and RM3, the leader provides better after-sales service.

The role interaction of friend (RF) refers to the research of Williams and Spiro (1985), and there are three items to be measured, including RF1, the leader often greets the members in daily life, RF2, when the members encounter difficulties in life, the leader can actively provide some services and help, and RF3, the leader posts some interesting content, including text, pictures, and videos.

The community group-buying trust (CT) refers to the study of Bart et al. (2005), and there are three items to be measured, including CT1, I believe the products provided by community group-buying have quality assurance, CT2, I believe the information of community group-buying is reliable, and CT3, I believe there is no fraud in community group-buying.

The interpersonal trust (IT) refers to the study of Su et al. (2021), and there are three items to be measured, including IT1, in addition to the business relationship, I am familiar with the leader in my life, IT2, I believe the leader can treat each other sincerely, and IT3, I believe the leader will not infringe on my rights and privacy.

The lock-in purchasing intention from a fixed leader (LP) refers the study of Sun and Li (2018), and there are three items to be measured, including LP1, in the future, I will long-term buy products from a fixed leader, LP2, I will recommend my friends or family to buy products from the leader I know, and LP3, I don’t have intentions to buy products from other leaders.

### Data Collection

Due to the influence of the emergence and rebound of the epidemic in the recent 2 years, the community group-buying market in Fuzhou, Fujian Province has expanded rapidly. Many brands have appeared, including the PUPU Supermarket, Sincerity Optimization, Dingdong Shopping, Jingdong 7FRESH, etc. The community group-buying leaders have distributed many points in this area. Therefore, the community group-buying market in Fuzhou is of great representativeness. In this study, the residents of 20 districts in 5 municipal districts of Fuzhou were selected as the survey objects, and the offline questionnaire was distributed. The survey time was from January 01, 2022 to January 20, 2022. To ensure the quality of the survey sample, first, the questionnaire explains the purpose of this survey in the beginning sentences, and starts with a screening question, “Have you ever purchased products in the community group-buying in the last 6 months? Yes/no.” When the respondent answers “no,” the questionnaire is terminated. Then, the respondents answer some questions related to the measurement items. Finally, the respondents need to provide some basic information, such as gender, age, average monthly income, etc. In this survey, 25 questionnaires were collected from each community, and a total of 500 questionnaires were collected. After eliminating 70 incomplete and non-conforming questionnaires, 430 valid questionnaires were obtained, which can be used for structural verification, reliability, and hypothesis testing.

Table 1 illustrates the demographics of the 430 consumers. In these samples, 67.4% of the respondents were aged between 30 and 49, belonging to the young and middle-aged group. They are familiar with and frequently use the e-commerce platforms, and are the main force of consumption of the new retail e-commerce business. In addition, 63.3% of the respondents were women, and 75.1% of the respondents had a monthly income of 5,000 CNY or more. Therefore, the structure of respondents meets the objectives of this study.

**TABLE 1:** Sample characteristics (*N* = 430).

Item	Frequency	Percentage
**Gender**		
Male	158	36.7
Female	272	63.3
**Age**		
Under 18	0	0
18–29	98	22.8
30–39	163	37.9
40–49	127	29.5
50 or over	42	9.8
**Average monthly income**		
<2,500 RMB	21	4.9
2,501–3,000 RMB	29	6.7
3,001–5,000 RMB	57	13.3
5,001–8,000 RMB	175	40.7
>8,000 RMB	148	34.4

### Non-response Bias and Common Method Bias

We did a non-response bias test referencing Armstrong and Overton (1977). Specifically, we divided the 430 respondents evenly by time stamp into two groups (early and late). Independent sample *t*-test experimental design was selected to examine for significant differences between “the early group” and “the late group.” The final results showed no significant differences. Therefore, there was no non-response bias in this study.

We used the method of controlling the non-measurable potential method factor to test the common method biases of the sample data, that is, add the common method factor into the structural equation model as a latent variable, and then compare the change of the model fitness after adding the latent variable (Podsakoff et al., 2003). The results showed that the change of each fit index was less than 0.05, which met the standard of Podsakoff et al. (2003). Therefore, there is no serious common method bias problem.

## Data and Empirical Analysis Results

### Reliability and Validity Analysis

Before the hypothesis test, the confirmatory factor analysis (CFA) was performed on all latent variables using AMOS 26.0 to evaluate the reliability and validity of the measurement model. First, the factor measurement model has a good fitting degree (χ^2^ = 290.577; χ^2^/*df* = 2.421; *p* = 0.000; GFI = 0.932; AGFI = 0.903; CFI = 0.980; TLI = 0.974; RMSEA = 0.058). Therefore, we can further test the reliability and validity. Second, the reliability of the measurement model is judged by the standardized factor loading (Std.), square multivariate correlation (SMC), and composite reliability (CR) of five constructs. The results are shown in Table 2. From the table, the Std. values of the five constructs are in the range of 0.787 ∼ 0.942, greater than 0.70 (*p* < 0.000), the SMC values are greater than 0.50, and the CR values are greater than 0.70, which meets the evaluation criteria of Fornell and Larcker (1981). Therefore, the above results fully support the reliability of the measurement model. Next, the validity of the measurement model is judged by convergent validity and discriminant validity. According to the evaluation criteria of Fornell and Larcker (1981), the average variance extraction (AVE) values of all latent variables are greater than 0.50, indicating that the measurement model has good convergence validity. Finally, as shown in Table 3, it is found that the square root value of the AVE of most latent variables is greater than the Pearson correlation coefficient of variables. The Pearson correlation coefficients of LP–IT and LP–CT are greater than the square root value of the AVE of LP, which may be considered as that the items of latent variables of LP, IT, and CT were confused among respondents (Zait and Bertea, 2011; Rönkkö and Cho, 2020). To further test the discriminant validity between LP, IT, and CT variables, the Chi-squared test on the structures of LP and IT; and LP and CT, respectively, were conducted. The results of the two groups indicated that the Chi-squared differences were significant (*p* < 0.05), which met the evaluation criteria of Segars (1997), indicating that both LP and IT structures and LP and CT structures showed discriminant validity. Therefore, the measurement model has good discriminant validity.

**TABLE 2:** Reliability analysis of research scale.

		Parameter significance estimation				Item reliability		Composite reliability	Convergent validity
	Item	Unstd.	S.E.	*z*-value	*p*	Std.	SMC	CR	AVE
Role interaction of merchant	RM1	1.000				0.898	0.806	0.891	0.733
	RM2	0.996	0.045	22.314	***	0.879	0.773		
	RM3	0.870	0.044	19.704	***	0.787	0.619		
Role interaction of friend	RF1	1.000				0.895	0.801	0.905	0.761
	RF2	1.030	0.044	23.644	***	0.878	0.771		
	RF3	0.962	0.043	22.437	***	0.843	0.711		
Community group-buying trust	CT1	1.000				0.899	0.808	0.922	0.798
	CT2	0.948	0.035	27.184	***	0.913	0.834		
	CT3	0.957	0.038	25.154	***	0.868	0.753		
Interpersonal trust	IT1	1.000				0.863	0.745	0.941	0.842
	IT2	1.066	0.037	28.760	***	0.946	0.895		
	IT3	1.058	0.037	28.599	***	0.942	0.887		
Lock-in purchasing intention from a fixed leader	LP1	1.000				0.898	0.806	0.905	0.761
	LP2	0.972	0.043	22.717	***	0.849	0.721		
	LP3	0.988	0.042	23.423	***	0.870	0.757		

***p < 0.001.

**TABLE 3:** Validity analysis of measurement model.

	AVE	LP	IT	CT	RF	RM
LP	0.761	**0.872**				
IT	0.842	0.890	**0.918**			
CT	0.798	0.953	0.886	**0.893**		
RF	0.761	0.787	0.766	0.832	**0.872**	
RM	0.733	0.721	0.712	0.740	0.687	**0.856**

*The bold values in the table are the square root of AVE of the latent variables, and the value below the diagonal is the absolute value of the Pearson correlation coefficient of variables; LP denotes the lock-in purchasing intention from a fixed leader, IT denotes the interpersonal trust, CT denotes the community group-buying trust, RF denotes the role interaction of friend, and RM denotes the role interaction of merchant.*

### Model Checking and Path Analysis

To test the measurement model, AMOS 26.0 was used. The fit index of the model was within the acceptable range (χ^2^ = 208.551; χ^2^/*df* = 2.543; *p* = 0.000; GFI = 0.939; AGFI = 0.911; CFI = 0.981; TLI = 0.975; RMSEA = 0.060), indicating that the structural equation model had good fitness.

**FIGURE 2:**
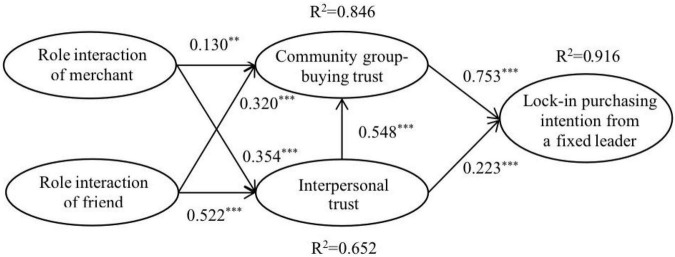
Path analysis diagram. ^**^p < 0.01, ^***^p < 0.001.

The test results of the model path and *R*^2^ measures are shown in Figure 2 and Table 4. First, in the context of community group-buying, the role interaction of merchant has a significantly positive influence on the community group-buying trust (β = 0.130; *p* = 0.002), and has a positive influence on the interpersonal trust (β = 0.354; *p* < 0.001). Therefore, we assume that Hypotheses H1a and H1b are supported. Meanwhile, the role interaction of friend has a significantly positive influence on the community group-buying trust (β = 0.320; *p* < 0.001), and has a positive influence on the interpersonal trust (β = 0.522; *p* < 0.001). Therefore, the data results support Hypotheses H2a and H2b. Second, interpersonal trust has a significantly positive influence on community group-buying trust (β = 0.548; *p* < 0.001), so we assume that Hypothesis H3 is supported. Finally, both community group-buying trust (β = 0.753; *p* < 0.001) and interpersonal trust (β = 0.223; *p* < 0.001) have a significantly positive influence on the lock-in purchasing intention from a fixed leader. Therefore, the data results support Hypotheses H4a and H4b.

**TABLE 4:** Fitting path test.

Path	Unstd.	*S.E.*	CR (*T*-value)	*p*	Std.	Hypothesis	Results
RM → CT	0.124	0.040	3.103	0.002	0.130	H1a	Supported
RM → IT	0.320	0.047	6.769	***	0.354	H1b	Supported
RF → CT	0.330	0.049	6.727	***	0.320	H2a	Supported
RF → IT	0.510	0.052	9.72	***	0.522	H2b	Supported
IT → CT	0.577	0.053	10.868	***	0.548	H3	Supported
CT → LP	0.744	0.068	10.971	***	0.753	H4a	Supported
IT → LP	0.232	0.067	3.454	***	0.223	H4b	Supported

(1) LP denotes the lock-in purchasing intention from a fixed leader, IT denotes the interpersonal trust, CT denotes the community group-buying trust, RF denotes the role interaction of friend, and RM denotes the role interaction of merchant. (2) ***p < 0.001.

### Test and Analysis Results of Mediating Effect

This study uses the Bootstrap method proposed by Hayes (2009) to analyze the mediating effect. The PROCESS macro in SPSS22.0 was used for verification. The results are shown in Table 5. From the table, the effect values (*t*) of the role interaction of merchant, the role interaction of friend, and the interpersonal trust on the lock-in purchasing intention from a fixed leader based on the mediating effect of community group-buying trust are 0.124 (*t* = 3.652), 0.199 (*t* = 5.192), and 0.624 (*t* = 15.291), respectively. There are no zero values in 95% of confidence intervals, indicating that the mediating effects are significant. The effect values of the role interaction of merchant and the role interaction of friend on the lock-in purchasing intention from a fixed leader based on the mediating effect of interpersonal trust are 0.097 (*t* = 3.129) and 0.085 (*t* = 2.294), respectively. Similarly, there are no zero values in 95% of confidence intervals, indicating that the mediating effects are significant.

**TABLE 5:** Results of mediating effect.

					Indirect effect		
	Total effect		Direct effect			Bootstrap 1,000 times 95% CI	
	β	*t*	β	*t*	β	BootLLCI	BootULCI
RM → CT → LP	0.124	3.652	0.052	1.748	0.072	0.020	0.137
RF → CT → LP	0.199	5.192	0.036	1.025	0.163	0.097	0.245
IT → CT → LP	0.624	15.291	0.322	7.551	0.301	0.222	0.394
RM → IT → LP	0.097	3.129	0.052	1.748	0.045	0.017	0.083
RF → IT → LP	0.085	2.294	0.036	1.025	0.049	0.017	0.092

*LP denotes the lock-in purchasing intention from a fixed leader, IT denotes the interpersonal trust, CT denotes the community group-buying trust, RF denotes the role interaction of friend, and RM denotes the role interaction of merchant.*

## Research Conclusion and Management Implications

Community group-buying based on acquaintance marketing has become a rapidly growing research field in the recent years, which needs more attention from enterprises and scholars. Based on the role theory and trust transfer theory, this study divided the role interactions of the leader into the following two types: Role interaction of merchant and role interaction of friend; and discussed the influence of multi-role interactions on consumers’ lock-in purchasing intention from a fixed leader. In addition, we also explored the intermediary role of the consumer trust. On that basis, the formation mechanism model of the lock-in purchasing intention from a fixed leader was constructed. Moreover, this study adopted a data sample composed of 430 consumers to conduct the empirical test. The results showed that the role interactions of merchant and friend can affect the interpersonal trust and community group-buying trust, and further enhance consumers’ lock-in purchasing intention from a fixed leader. The results of this study are of certain theoretical and management significance for the development of community group-buying.

### Discussion of Research Conclusion

(1) In the context of community group-buying, both role interactions of merchant and friend can positively affect the community group-buying trust, which supports the Hypotheses H1a and H2a. Compared with Hypothesis H1a, the results of Hypothesis H2a are more significant (β = 0.13^**^ < β = 0.320^***^), which can further distinguish the influence of the group-buying leader according to different role interactions. This result confirms that the interaction of the group-buying leader with the role of merchant or friend based on the behavior pattern that matches their role expectation can have differences in the establishment of community group-buying trust (Zhang et al., 2018; Li et al., 2020), and the role interaction of friend has a greater impact on the establishment of platform trust. In addition, interpersonal trust also has a positive impact on community group-buying trust, which supports Hypothesis H3 and verifies the transfer effect of trust between group-buying leaders and platform. In general, when Hypotheses H1a, H2a, and H3 are considered simultaneously, the interpersonal trust has the greatest influence on community group-buying trust, β = 0.5428^***^. This research result can be attributed to the significant influence that the interpersonal trust imposed on the community group-buying trust, thereby reducing the direct effect of the interactions of merchant and friend. In addition, since the category of community group-buying is mainly fresh agricultural products, compared with standardized products, consumers may worry about the quality of the products. Therefore, based on the interpersonal trust in the leader, consumers can reduce their worries about the product and purchase without hesitation. Conclusively, interpersonal trust and the role interaction of friend are important conditions affecting community group-buying trust and the accomplishment of the whole transaction. This is also consistent with the viewpoint of Zhuang and Xi (2003) and Fu et al. (2015) that the “relationship” goes before transaction in the marketing of China.
(2) In the context of community group-buying, the antecedents of interpersonal trust, the role interaction of merchant, and the role interaction of friend can positively affect the interpersonal trust, which supports Hypotheses H1b and H2b. The regression results of this study show that the role interaction of friend affects the path coefficients greater on interpersonal trust than the role interaction of merchant, i.e., β = 0.354^***^ < β = 0.522^***^. These results have confirmed the research of Huang and Ha (2020), that is, warm-oriented service interaction strategies are more likely to alleviate the response of online consumers to complaints. The reason for this result may be that compared with the role interaction of merchant which focuses on providing information and displaying ability, the role interaction of friend is usually in the form of mutual assistance, goodwill, and recommendation in community group-buying. Since consumers are living in the same community or nearby, the human resources accumulated in offline interaction can effectively maintain interpersonal trust. This is also in line with the special relationship operation mechanism of “human debt” in China’s social communication (Zhai, 2021).
(3) Considering the particularity of the relationship between the group-buying leader and neighbors in the community, trust can be divided into community group-buying trust and interpersonal trust. Interpersonal trust in the group-buying leader is a special trust dimension in this situation. However, most studies on group-buying only consider the trust in the platform and ignore interpersonal trust (Wang et al., 2016). The results show that the community group-buying trust and interpersonal trust have different degrees of positive influences on consumers’ lock-in purchasing intention from a fixed leader, which supports Hypotheses H4a and H4b. This shows that it is necessary to divide trust into community group-buying trust and interpersonal trust. Interestingly, in this study, the community group-buying trust is more than three times as much as the interpersonal trust, i.e., 0.753 > 0.223. Consumers if only based on interpersonal trust will impose less direct impact on the lock-in purchasing intention from a fixed leader, which is different from the conclusion of the previous research that trust between buyers and sellers is easy to be converted into purchase intention (Geng, 2021; Li and Li, 2022). This result may be due to the influence of the mediating effect of the community group-buying trust on the interpersonal trust on consumers’ lock-in purchase intention from a fixed leader to a certain extent, and further reveals that interpersonal trust may not be as direct and important in platform transactions as in traditional transactions in the past. Specifically, although interpersonal trust brings some trading advantages to the leader, due to the fierce market competition, there are many leaders of different platforms in the same community, and consumers will not put too much emotion in the same leader. Instead, they will compare and screen among different leaders, and finally buy the products according to the quality, price, and other factors.

### Theoretical Contribution

Establishing a good interaction relationship with consumers is the focus of online retailers, and the interactive marketing strategy is also the focus of scholars at home and abroad. The previous research mainly studied from the perspectives of interaction content (KöHler et al., 2011) and the disclosure degree of persuasion intention of the interaction content (Zhu et al., 2020), and few scholars combined with the identity of merchants to make a division according to the interaction angle of different roles. In social e-commerce, the coexistence of the roles of friend and merchant imposes more impact on interactive marketing. Therefore, based on the role theory, this study emphasizes the importance of different roles of the community group-buying leader, and reveals that different forms of interactions between buyers and sellers can promote consumer trust to a certain extent from role interactions of friend and merchant, thereby leading to the consumers’ long-term purchase intention to a fixed leader. This research conclusion explores the influence path of multi-role interaction strategies on the decision-making behavior of consumers’ lock-in purchasing intention, provides a new theoretical explanation for the development of community group-buying based on acquaintance marketing in reality, and further enriches and expands the application of role theory in the purchase decision making of consumers.

In the previous studies, consumer trust was taken as a single dimension variable of overall trust for measurement. For example, the characteristics of online and offline combination in community group-buying directly affect consumers’ perception of trust (Li et al., 2020); the fast relationship established by the social media interactivity (online comments and instant messaging) can enhance the consumer trust (Chong et al., 2017). However, in the community group-buying situation, the transaction process involves the following three parties: The intermediary platform, the group-buying leader who provides offline services, and consumers (Hwang and Kim, 2017). Therefore, consumer trust is no longer based on the overall perception but also the judgment on the interpersonal trust of the group-buying leader and the trust in the community group-buying platform. Therefore, based on the interaction of double roles, this study introduces different dimensions of the consumer trust (i.e., community group-buying trust and interpersonal trust) as mediators to deeply explore the influence mechanism of multi-role interactions of the merchant on the consumers’ lock-in purchasing intention. Interestingly, it can be found that there is a significant difference between the community group-buying trust and interpersonal trust. The community group-buying trust has a greater weight than interpersonal trust, which means that when consumers decide whether to long-term buy products for a fixed leader, they will be more inclined to seriously consider the overall quality of community group-buying products, services, systems, and so on. This finding helps to expand the relevant research of trust transfer theory, that is, the community group-buying trust has a mediating effect on the influence of interpersonal trust on consumers’ lock-in purchasing intention from a fixed leader.

### Management Implications

In the recent years, community group-buying has developed rapidly due to its advantages of low-cost structure, high-speed trading, high transparency, and large-scale, which has also effectively improved the well-being of consumers. However, in practice, many enterprises still use the mode of “price war” to attract consumers (Duan, 2022), and ignore the important “bridge” role of the leader. They even have no idea of the internal mechanism and influencing factors of how to enhance the stickiness of consumers. Therefore, the conclusion of this study can provide guidance for enterprises and leaders on how to implement interactive marketing strategies with consumers, mainly including the following two aspects:

(1) The interaction between the community leader and members can stimulate the consumer trust to a certain extent. Compared with the role interaction of merchant, the role interaction of friend has an important influence on the community group-buying trust and interpersonal trust. Therefore, it is necessary for the leader to change the original traditional role identity of merchant, make full use of the advantages of offline communities, and express more care and sincerity in the role of friend. For example, give generous assistance to the community residents in need of help, provide some personalized services to the residents, create a happy interactive atmosphere, and guide the high-frequency interaction among the members, thereby narrowing the emotional distance, constantly strengthening the recognition of the role of friend of the leader, and enhancing the trust.
(2) Facing the highly competitive market, consumers’ lock-in purchasing behavior is the basis for the long-term survival of capital enterprises and the leaders to a certain extent. The continuous trading relationship between the leader and members can be continued based on the emotional relationship. This study shows that compared with interpersonal trust, consumer trust in the community group-buying has a greater influence on consumers’ lock-in purchasing intention from a fixed leader. Therefore, from the perspective of leaders, only establishing simple interpersonal trust with consumers is difficult for the leader to effectively stimulate consumers’ willingness to lock a fixed leader for purchasing. To make consumers believe the community group-buying, the leader also needs to transmit and display the information about the production and purchase process and the attribute characteristics of the platform products to consumers, provide some reliable after-sales services in time to eliminate consumers’ concerns about the risk of online shopping, and establish trust between consumers, products and platforms, thereby advancing the better development of community group-buying. From the perspective of enterprises, community group-buying enterprises also need to pay attention to the trust transfer effect from interpersonal trust to the trust in community group-buying. Meanwhile, it is needed for enterprises to transfer this trust to the platform by strengthening the rational judgment of consumers on the group-buying leader, which can avoid the risk of the loss of consumers caused by the loss of the group-buying leader, and finally realize the retention of consumers.

### Limitations and Future Research Directions

First, this study used cross-sectional data to verify the hypothetical model, and it was not possible to compare respondent demographics with the overall population. Thus, other methods such as longitudinal data and experimental methods can be used to verify the conclusions of this study in future research.

Second, the boundary conditions of the model in the process of trust transfer between interpersonal trust and community group-buying trust have not been investigated in this study. For example, the mutually beneficial belief is considered to be an important component of relationship quality, and the existence of mutually beneficial belief may be conducive to strengthening the process of trust transfer. Therefore, the impact of relevant regulatory variables should be studied in future research.

Third, due to the particularity of the double-role identity of the leader, the interaction mode can not only have a positive impact on business performance, but also may have a negative impact. Especially when consumers have the perception of role conflict, it may stimulate the resistance of consumers. Therefore, it can be further studied from the perspective of role conflict in the future.

## Data Availability Statement

The original contributions presented in the study are included in the article/supplementary material, further inquiries can be directed to the corresponding author.

## Author Contributions

JW, YC, HP, and AX contributed to conception and design of the study. JW wrote the first draft of the manuscript and performed the statistical analysis. YC, HP, and AX organized the database and wrote sections of the manuscript. All authors contributed to manuscript revision, read, and approved the submitted version.

## Conflict of Interest

The authors declare that the research was conducted in the absence of any commercial or financial relationships that could be construed as a potential conflict of interest.

## Publisher’s Note

All claims expressed in this article are solely those of the authors and do not necessarily represent those of their affiliated organizations, or those of the publisher, the editors and the reviewers. Any product that may be evaluated in this article, or claim that may be made by its manufacturer, is not guaranteed or endorsed by the publisher.
